# The Performance of Deep Learning Algorithms on Automatic Pulmonary Nodule Detection and Classification Tested on Different Datasets That Are Not Derived from LIDC-IDRI: A Systematic Review

**DOI:** 10.3390/diagnostics9040207

**Published:** 2019-11-29

**Authors:** Dana Li, Bolette Mikela Vilmun, Jonathan Frederik Carlsen, Elisabeth Albrecht-Beste, Carsten Ammitzbøl Lauridsen, Michael Bachmann Nielsen, Kristoffer Lindskov Hansen

**Affiliations:** 1 Department of Diagnostic Radiology, Copenhagen University Hospital, Rigshospitalet, 2100 Copenhagen, Denmark; bolette.mikela.vilmun.01@regionh.dk (B.M.V.); jonathan.frederik.carlsen@regionh.dk (J.F.C.); carsten.ammitzboel.lauridsen.01@regionh.dk (C.A.L.); mbn@dadlnet.dk (M.B.N.); Kristoffer.Lindskov.Hansen.01@regionh.dk (K.L.H.); 2 Department of Clinical Medicine, University of Copenhagen, 2100 Copenhagen, Denmark; 3 Department of Clinical Physiology, Nuclear Medicine and PET, Copenhagen University Hospital, Rigshospitalet, 2100 Copenhagen, Denmark; elisabeth.albrecht-beste@regionh.dk; 4 Department of Technology, Faculty of Health and Technology, University College Copenhagen, 2200 Copenhagen, Denmark

**Keywords:** deep learning, nodule detection, nodule classification, artificial intelligence

## Abstract

The aim of this study was to systematically review the performance of deep learning technology in detecting and classifying pulmonary nodules on computed tomography (CT) scans that were not from the Lung Image Database Consortium and Image Database Resource Initiative (LIDC-IDRI) database. Furthermore, we explored the difference in performance when the deep learning technology was applied to test datasets different from the training datasets. Only peer-reviewed, original research articles utilizing deep learning technology were included in this study, and only results from testing on datasets other than the LIDC-IDRI were included. We searched a total of six databases: EMBASE, PubMed, Cochrane Library, the Institute of Electrical and Electronics Engineers, Inc. (IEEE), Scopus, and Web of Science. This resulted in 1782 studies after duplicates were removed, and a total of 26 studies were included in this systematic review. Three studies explored the performance of pulmonary nodule detection only, 16 studies explored the performance of pulmonary nodule classification only, and 7 studies had reports of both pulmonary nodule detection and classification. Three different deep learning architectures were mentioned amongst the included studies: convolutional neural network (CNN), massive training artificial neural network (MTANN), and deep stacked denoising autoencoder extreme learning machine (SDAE-ELM). The studies reached a classification accuracy between 68–99.6% and a detection accuracy between 80.6–94%. Performance of deep learning technology in studies using different test and training datasets was comparable to studies using same type of test and training datasets. In conclusion, deep learning was able to achieve high levels of accuracy, sensitivity, and/or specificity in detecting and/or classifying nodules when applied to pulmonary CT scans not from the LIDC-IDRI database.

## 1. Introduction

Lung cancer is still the leading cause of cancer-related deaths in both the United States [1] and Europe, where it accounts for 20.9% of all cancer-related deaths [2]. Because of this, efforts have been made to reduce the incidence of lung cancer, primarily through the promotion of smoking cessation and lung cancer screening of high-risk individuals. Although much has been done with prevention, there are still around 370,000 new cases of lung cancer each year [2]. It is therefore crucial to diagnose lung cancer at an early stage to increase patients’ chance of survival. 

Early efforts to detect lung cancer through imaging were widely investigated, and no significant reduction in mortality by screening with traditional chest radiography was reported [3,4]. Since then, computed tomography (CT) has emerged as an imaging method with superior sensitivity in detecting lung nodules, and screening with CT has been shown to be superior to traditional chest radiography in reducing mortality from lung cancer [5]. When chest radiographs are replaced by CT scans for pulmonary cancer assessment, there will inevitably be an increase in workload for the radiologists, which results in missed cases and errors in diagnostics [6,7]. 

To aid radiologists in more accurate and time-efficient detection and diagnosis of pulmonary nodules, several computer-aided diagnosis and detection schemes have been developed [8,9,10]; the best known computer-aided diagnosis schemes to distinguish between benign and malignant nodules are based on volume doubling time [11]. Recently, deep learning has emerged as a more intelligent and accurate image classification technology [12] and has been adapted to classify medical images including chest CTs [13,14]. To the best of our knowledge, deep learning technology has yet to be successfully implemented in an everyday clinical workflow when diagnosing pulmonary nodules. A reason for this may be that deep learning algorithms need to be trained on data that are similar to the final task data [15]. Most studies have trained and tested their algorithms on the large and publicly available Lung Image Database Consortium and Image Database Resource Initiative (LIDC-IDRI) dataset, which makes the studies homogenous [16]. Few studies have tested their algorithms on datasets not from LIDC-IDRI, and only a subgroup of those have trained their algorithms on datasets that were not obtained the same way as the final test data [17,18]. 

The study aim of this systematic review was to investigate how deep learning performs for pulmonary nodule detection and/or classification of CT scans when the method is tested on datasets that are not from LIDC-IDRI. Furthermore, the study aim was to investigate whether the performance of deep learning is reduced when the algorithm is tested on a dataset that is different from the training dataset.

## 2. Materials and Methods

### Literature Search Strategy

The literature search was completed on 27 May 2019 from six databases: EMBASE, PubMed, Cochrane Library, the Institute of Electrical and Electronics Engineers, Inc. (IEEE), Scopus, and Web of Science. The search was restricted to peer-reviewed publications of original research written in the English language and published in the 10 years preceding the search completion date. 

The following specific MESH terms in PubMed were used: “lung”, “respiratory system”, “classification”, “artificial intelligence”, “tomography, emission-computed”, “tomography”, “X-ray”, and “tomography scanners, X-ray computed”. 

The terms were then combined with following text words in the title and/or abstract: “lung”, “pulmonary”, “respiratory”, “classification”, “characterization”, “detection”, “artificial intelligence”, “machine learning”, “deep learning”, “neural network”, “computer-assisted”, “computer-aided”, “CT”, and “computed tomography”. To perform the search in EMBASE, the following combinations of EMTREE terms and text words were used: (Classification (EMTREE term) OR sensitivity and specificity (EMTREE term) OR accuracy (EMTREE term) OR diagnostic accuracy (EMTREE term) OR diagnostic test accuracy study (EMTREE term) OR diagnostic reasoning (EMTREE term) OR “detection” OR “classification” OR “diagnosis”) AND (artificial intelligence (EMTREE term) OR artificial neural network (EMTREE term) OR machine learning (EMTREE term) OR computer assisted diagnosis (EMTREE term) OR “neural network” OR “deep learning”) AND (lung (EMTREE term) OR “pulmonary”) AND (whole body CT (EMTREE term) OR computer assisted tomography (EMTREE term) OR “CT” OR “computed tomography” OR “computer tomography”).

After removal of duplicates, all titles and abstracts retrieved from the searches were independently screened by two authors (DL and BMV). If the two authors could not reach an agreement on a study, a third author (JFC) assessed and resolved the disagreement. Data were extracted by DL and BMV via of pre-piloted forms. To describe the performance of the proposed deep learning algorithms on detection and/or classification of pulmonary nodules, we used a combination of narrative synthesis and compared measures of sensitivity, specificity, area under the curve (AUC), and accuracy if these were available. If information from a confusion matrix was available, sensitivity and specificity were independently calculated by DL and double-checked by BMV.

## 3. Study Inclusion Criteria

Peer-reviewed original research articles published after 2009 were reviewed for inclusion in this systematic review. Studies that examined the use of machine learning in detection and/or classification of pulmonary nodules were selected

1 If the technology was based on deep learning or had primary components of deep learning algorithms used to either detect pulmonary nodules and/or classify these nodules into different categories,
  and
2 if the deep learning algorithm was tested on CT scans that were not part of or derived from the LIDC-IDRI database,
  and
3 if any performance measures were reported, preferably in the form of, but not limited to sensitivity, specificity, accuracy, and/or AUC.

If more than one algorithm based on the same type of deep learning architecture was tested in the same study, the best performing algorithm was chosen for the results. Datasets were defined as different if the included CT images were obtained from different hospitals/locations/types of databases. Unless otherwise stated, the CT images used in the training dataset were not a part of the test dataset.

## 4. Literature Search Results

**Figure 1:**
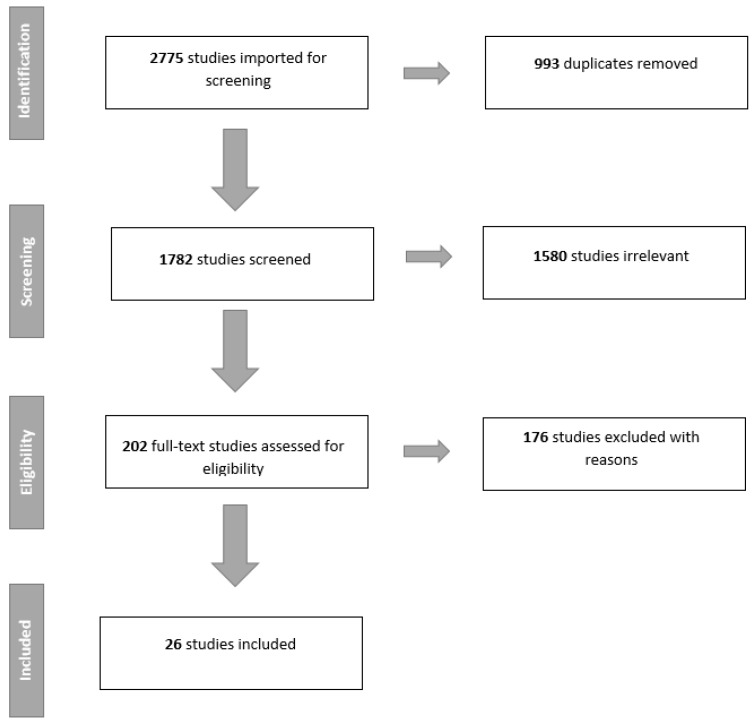
Preferred reporting items for systematic reviews and meta-analyses (PRISMA) flowchart of the literature search and study selection.

A total of 26 studies were included in this review. Due to the heterogeneity of the results from the different studies, it was not possible to perform a meta-analysis. Figure 1 summarizes the study selection as a PRISMA flowchart. Ten studies investigated the use of deep learning for nodule detection (Table 1), i.e., nodule or non-nodule, and 23 studies examined classification performance of nodules (Table 2). Seven studies reported results on both detection and classification performance. Table 3 shows the performance of the different algorithms for nodule classification when arranged after specific types of performance measurements.

Three different deep learning algorithms were mentioned in the studies: convolutional neural network (CNN), massive training artificial neural network (MTANN), and deep supervised denoising autoencoder architecture based on extreme learning machine (SDAE-ELM). CNN and MTANN are both end-to-end machine-learning algorithms, meaning that inputs are complete pixelated images and are processed without known components of specific feature detection and trained using backpropagation. MTANN outputs an image with the likelihood of it being a certain class, while CNN usually outputs results in class categories instead of images [43]. The advantage of MTANN is fewer training cases compared to CNN without compromising classification performance [20]. SDAE-ELM is a feature vector deep learning algorithm combined with ELM, which is a feed-forward neural network [37]. The advantages of stacked autoencoders include fewer training cases compared to, for example, CNN, since stacked autoencoders are able to generate new images from the image characteristic feature vectors [44]. 

## 5. Detection Only (3 Studies)

Setio et al. [18] and Liu et al. [24] both proposed CNN-based algorithms for pulmonary nodule detection. Setio et al. [18] tested their CNN-based program (ConvNets) on cases from the Danish Lung Cancer Screening Trial (DLCST), while Liu et al. [24] tested their algorithm on the Kaggle Data Science Bowl 2017 (DSB17) [45]. A third study by Wang et al. [26] tested their faster region-CNN (RCNN) based program on cases from an independent database and achieved 75.6% sensitivity on nodule detection. All studies reached a sensitivity between 75.6–85.6%. Only Setio et al. [18] published an accuracy rate, which was 94% (Table 1).

Setio et al. [18] trained and tested their algorithm on different types of datasets and achieved a sensitivity of 76.5%, while Liu et al. [24] and Wang et al. [26] both tested and trained their algorithm on the same type of dataset and achieved a sensitivity of 75.6% and 85.6%, respectively (Table 1 and Table 3).

## 6. Classification Only (16 Studies)

For studies that only reported results on classification performance, five studies [34,35,37,40,42] tested on local, independently obtained datasets. All studies provided reports of accuracy, which ranged between 68–92%. Four of these studies [34,35,40,42] had deep learning architectures based on CNN, while only Qiang et al. [37] used SDAE-ELM. For Nishio et al. [34], sensitivity and specificity were calculated from values given in a confusion matrix for benign, primary cancer, and metastatic cancer as 50.1% and 84.4%, 77.6% and 77.4%, and 74% and 88.2%, respectively. Onishi et al. [35] had an overall classification accuracy of 81.7%. The rest of the studies [37,40,42] categorized their nodules into malign or benign types and reached a sensitivity between 84.4–96% (Table 2).

Four studies [31,32,33,41] tested their CNN-based algorithm on the Early Lung Cancer Action Program (ELCAP) public lung database [46]. Besides Liu et al. [32], who did not provide reports on accuracy, the other studies [31,33,41] reached classification accuracies between 90.3–94.5%. Both Liu et al. [33] and Yuan et al. [41] classified nodules into multiple categories and calculated the proportion of a specific nodule type, e.g., the proportion of classified well-circumscribed nodules actually well circumscribed, which was 95.0% for Liu et al. and 96.1% for Yuan et al. Lakshmanaprabu et al. [31] tested whether different CT images were categorized correctly as to whether an image was normal or contained malign or benign nodules; results are displayed in Table 2. 

Three studies [27,36,39] reported classification results tested on the DSB17 dataset [45]. They were all CNN-based algorithms testing whether a patient had cancer or no cancer without testing the individual nodule. They reached accuracy levels between 86.6–91.8%. Other studies that reported results on classification only tested on a variety of dataset types. All had algorithms based on CNN architecture. Ciompi et al. (2015) [28] tested on CT scans from the Dutch–Belgian Randomized Lung Cancer Screening Trial (Dutch acronym; NELSON [47] and, in a later study [29], they tested for solid (recall; 82.2%), non-solid (recall; 87.4%), part-solid (recall; 64.9%), calcified (recall; 82.8%), peri-fissural (recall; 60.4%), and spiculated nodules (recall; 64.3%) on patients from the DLCST.

Jakimovski and Davcev [30] used an algorithm that was both trained and tested on the Image and Data Archive of the University of South Carolina and Laboratory of Neuro Imaging (LONI database) [48] and achieved an accuracy of 99.6%, a sensitivity of 99.9%, and specificity of 98.6% for their best-performing algorithm. The algorithm from Jakimovski et al. [30] outputted a single decimal value between 0.0 and 1.0, where 0.0 was not cancer and 1.0 was cancer. They converted the value to a percentage and set a minimal threshold value at 73% before the image was categorized as cancer. The output was matched to the original database results classified by medical personnel as cancerous or not based on lung tissue biopsy [48]. Rangaswamy et al. [38] trained and tested three different classifiers on the publicly available database of interstitial lung disease (ILD) [49] and categorized the CT images into whether or not they contained malign or benign nodules. They found that CNN achieved the best classification result compared to the other classifiers used and achieved an accuracy of 96% (Table 2).

For the above-mentioned studies, which only investigated classification performance, four studies [29,33,41,42] trained and tested on different types of datasets and achieved accuracies between 79.5–93.6%. The rest of the studies [27,28,30,31,32,34,35,36,37,38,39,40] trained and tested on the same types of datasets and achieved accuracies between 68–99.6% (Table 2 and Table 3). 

## 7. Both Detection and Classification (7 Studies)

Five studies [17,19,20,22,25] had results on both classification and detection and tested on local, independently obtained datasets. While all the studies tested a CNN architecture, Tajbakhsh and Suzuki [20] tested both CNN- and MTANN-based algorithms. Three of the studies [17,19,22] measured detection performance using sensitivity and they reached levels between 86.2–97% (Table 1). Tajbakhsh and Suzuki [20] collected information of false positives when 100% sensitivity was achieved with MTANN and CNN, which resulted in 2.7 and 22.7 false positives per patient, respectively. Detection performance was measured by Wang et al. [25] using the kappa consistency coefficient and reached 0.94 when compared to human experts. On classification, four of the above-mentioned studies [19,20,22,25] tested on dichotomous categories. Two of the studies [20,25] reported AUC values of 77.6% and 90.6%. Chen et al. [22] achieved an overall classification accuracy of 87.5% when classifying adenocarcinomas and benign nodules, and Suzuki [19] achieved 96% sensitivity when classifying malign nodules (Table 2). Li et al. [17] tested the performance of characterizing nodules into three pulmonary nodule categories: solid (sensitivity: 90.3%; specificity: 100%), part-solid (sensitivity: 55.5%; specificity: 93%), and ground glass types (sensitivity: 100%; specificity: 96.1%).

The rest of the studies [21,23] tested on different types of datasets. Liao et al. [23] tested on data from DSB17 [45], while Masood et al. [21] tested on four different types of datasets for pulmonary nodule detection and on independently obtained data for classification performance. On detection, they reached a sensitivity of 85.6% and 74.6% (Table 1). Liao et al. [23] classified data into dichotomous categories, while Masood et al. [21] classified pulmonary nodules into four nodule stages. They reached classification accuracies of 81.4% and 96.3%, respectively (Table 2). 

On detection, two studies [17,19] tested and trained on different types of datasets and achieved sensitivities of 86.2% and 97.0%, while the studies that trained and tested on the same types of dataset [20,21,22,23,25] had sensitivities between 74.6–97% (Table 1). On classification, the two studies [17,19] that trained and tested on different types of dataset achieved sensitivities of 96% and 100%, and the studies that trained and tested on the same types of dataset [20,21,22,23,25] achieved sensitivities between 76.5–83.7% and accuracies between 81.4–96.3% (Table 2 and Table 3).

## 8. Discussion

We found a total of 26 studies that tested deep learning algorithms on datasets that were not from the LIDC-IDRI database. Of these studies, 27% (*n* = 7) tested their algorithms on datasets that were different from training datasets. We found that for testing diagnostic accuracy of pulmonary nodules on CT scans, CNN was the preferred deep learning architecture, followed by MTANN and deep SDAE-ELM.

Several other studies have trained and tested deep learning algorithms on the large, publicly accessible LIDC-IDRI database [16] and, recently, a systematic review was published overviewing the different studies that have tested on this database [50]. However, to review deep learning performance it is also necessary to review studies that did not use the LIDC-IDRI, as CT scans may vary from region to region. Hence, in this paper, only studies not using the LIDC-IDRI were included.

Algorithms with CNN architecture reached accuracies between 68–99.6% (Table 2) on classification and 80.6–94% (Table 1) on detection. Compared to a previous study using CNN-based algorithms on CT scans from the LIDC-IDRI [50], there was no observed difference in classification accuracy. Sensitivity and specificity for classification found in this review were between 76.5–99.9% and 80.1–98.7% (Table 2), respectively, which are also comparable to results of the CNN-based algorithms tested on the LIDC-IDRI [50]. Only Li et al. [17], who trained their algorithm on the LIDC-IDRI but tested on an independent dataset, had a noticeably low sensitivity result when classifying part-solid nodules (55.5%), and their algorithm was generally outperformed by double reading by radiologists on all categories (solid, part-solid, and ground glass).

MTANN reached a sensitivity of 97–100% on nodule detection (Table 1) and an AUC of 77.6–88.1% on classification (Table 2). This was generally higher than the sensitivity results reached by CNN for detection (74.6–97%) and classification AUC (78–90.6%). Some studies explored the difference in detection and classification performance between MTANN and CNN, and generally found MTANN to perform better than CNN [20,51]. One study [52] found that MTANN required much fewer training data compared to CNN, which could lead to a faster implementation of deep learning technology in a clinical setting, since fewer resources have to be allocated for training. Further investigations of MTANN as a pulmonary nodule diagnosis system are required, since CNN is still the most frequently used deep learning architecture for pulmonary nodule diagnosis [50]. 

We only found one study [37] that used an architecture other than MTANN or CNN. Qiang et al. [37] proposed a lung nodule classification system based on deep SDAE-ELM. The results were comparable to results obtained by CNN- and MTANN-based algorithms. To the best of our knowledge, no other study has yet investigated the deep SDAE-ELM architecture for pulmonary nodule diagnostics in CT images.

The two main issues with deep learning in imaging diagnostics are small training datasets and overfitting. To prevent the algorithm from overfitting, e.g., diagnosing background noise to be something of importance, more training data are required, which can be cumbersome in a clinical setting [53]. Studies have therefore examined transferability in deep learning, and some studies suggest that test data should be similar to training data for improved recognition results [15]. 

In our study, no tendency of reduced performance was observed for the algorithms trained and tested on different datasets compared to the algorithms tested and trained on the same type of dataset. When classification performance was measured using sensitivity (Table 3a), studies that used same type of dataset for test and training ranged between 76.5–99.9%, while the two studies [19,42] that tested and trained on different types of datasets had a sensitivity of 96%. We found no studies that trained and tested on different types of datasets measuring performance in AUC (Table 3b). Accuracy results for studies that tested and trained on same type of dataset were between 68–96.3%, while accuracy results from studies that tested and trained on different types of datasets were between 79.5–93.9% (Table 3c). All studies reported sensitivity of detection. Sensitivity ranged from 74.697% for studies tested and trained on same type of dataset, and from 76.6–97% for studies tested and trained on different types of dataset (Table 1). Our findings were in accordance with previous studies and suggests that comparable results can be reached despite datasets being of different patient composition and scan parameters, as long as they are similar in the underlying category and source type, e.g., lung nodule detection and CT [54]. Because of this tendency, studies have had success with training their algorithms through pre-training [55], transfer learning [56], and/or fine-tuning [57] to bypass the problem of a small training dataset, in addition to developing variations of algorithms that are based on other deep learning technologies besides the popular CNN, e.g., MTANN and deep SDAE-ELM. 

The heterogeneity of the included studies was a limitation of this review, since this prevented us from performing a meta-analysis to statistically compare the performance of deep learning algorithms. Thus, our study could not conclude whether there was a statistically significant difference in the performance of detection and/or classification by deep learning when trained and tested on the same or on different types of datasets. There may also be a risk of publication bias in these types of studies, since it may not seem relevant for the authors to submit research for publication with low or negative results of their algorithm. However, our study strengths include many studies from a variety of literature search engines and a systematic literature search ensuring that no relevant studies were missed. 

Several large companies have invested in researching deep learning in general image recognition of day-to-day objects [58,59] and, recently, some vendors have moved towards automatic recognition in clinical radiology [60]. With the increasing popularity of artificial intelligence emerging in healthcare and the increasing workload for radiologists, it would be wise to implement deep learning in clinical practice, but, to the best of our knowledge, there has not been any consistent, standardized incorporation of deep learning into the workflow of clinical radiology for pulmonary nodules. The next step should be to move forward with research on the clinical applications and use of deep learning in medical imaging and day-to-day workflow. 

## 9. Conclusions

Studies on deep learning found high levels of accuracy, sensitivity, and/or specificity in detecting and/or classifying pulmonary nodules on CT scans that were not from the LIDC-IDRI database. A tendency of comparable performance levels was observed regardless of whether the deep learning algorithms were trained and tested on the same type of dataset or on different types of dataset. To aid radiologists in their diagnostic work, artificial intelligence will become a valuable tool in the future, providing more accurate and time-efficient detection and diagnosis of pulmonary nodules; however, more studies and development are warranted.

## Figures and Tables

**Table 1:** Performance of the studies exploring detection of pulmonary nodules.

Detection								
Author	Year	Deep Learning Architecture	Dataset for Training	Dataset for Testing	Sensitivity	Specificity	AUC	Accuracy
Suzuki, Kenji * [19]	2009	MTANN	Independent dataset A	Independent dataset B	97	N/A	N/A	N/A
Tajbakhsh, Nima et al. [20]	2017	CNN	Independent dataset	Independent dataset	100	N/A	N/A	N/A
		MTANN	Independent dataset	Independent dataset	100	N/A	N/A	N/A
Masood, Anum et al. [21]	2018	FCNN	LIDC-IDRI, RIDER, LungCT-diagnosis, LUNA16, LISS, SPIE challenge dataset and independent dataset	RIDER	74.6	86.5	N/A	80.6
				SPIE challenge dataset	81.2	83	N/A	84.9
				LungCT-diagnosis	82.5	93.6	N/A	89.5
				Independent dataset	83.7	96.2	N/A	86.3
Chen, Sihang et al. [22]	2019	CNN	Independent dataset	Independent dataset	97	N/A	N/A	N/A
Liao, Fangzhou et al. [23]	2019	CNN	LUNA16 and DSB17	DSB17	85.6	N/A	N/A	N/A
Liu, Mingzhe et al. [24]	2018	CNN	LUNA16 and DSB17	DSB17	85.6	N/A	N/A	N/A
Li, Li et al. * [17]	2018	CNN	LIDC-IDRI and NLST	Independent dataset	86.2	N/A	N/A	N/A
Wang, Yang et al. [25]	2019	RCNN	Independent dataset	Independent dataset	N/A	N/A	N/A	N/A
Setio, A.A.A et al. * [18]	2016	CNN	LIDC-IDRI and ANODE09	DLCST	76.5	N/A	N/A	94
				ANODE09	N/A	N/A	N/A	N/A
Wang, Jun et al. [26]	2019	CNN	Tianchi AI challenge dataset and independent dataset	Independent dataset	75.6	N/A	N/A	N/A

Studies marked with * are studies where test dataset was different from training dataset. AUC: area under the curve. Abbreviations: massive training artificial neural network (MTANN), convolutional neural network (CNN), lung image database consortium and image database resource initiative (LIDC-IDRI), reference image database to evaluate therapy response (RIDER), Society of Photo-Optical Instrumentation Engineers (SPIE), lung nodule analysis 2016 (LUNA16), lung CT imaging signs (LISS), Kaggle data science bowl 2017 (DSB17), Danish lung cancer screening trial (DLCST), automatic nodule detection 2009 (ANODE09).

**Table 2:** Performance of studies exploring classification of pulmonary nodules.

Classification									
Author	Year	Deep Learning Architecture	Dataset for Training	Dataset for Testing	Categories for Testing	Sensitivity	Specificity	AUC	Accuracy
Alakwaa, Wafaa et al. [27]	2017	CNN	LUNA16 and DSB17	DSB17	Cancer vs. no cancer	N/A	N/A	N/A	86.6
Chen, Sihang et al. [22]	2019	CNN	Independent dataset	Independent dataset	Adenocarcinoma vs. benign	N/A	N/A	N/A	87.5
Ciompi, Francesco et al. [28]	2015	CNN	ImageNet and NELSON	NELSON	Peri-fissural nodules (PFN) vs. non-PFN	N/A	N/A	84.7	N/A
Ciompi, Francesco et al. *[29]	2017	CNN	MILD	DLCST	Multiple categories (overall)	N/A	N/A	N/A	79.5
Jakimovski, Goran et al. [30]	2019	CDNN	LONI database	LONI database	Cancer vs. no cancer	99.9	98.7	N/A	99.6
Lakshmanaprabu, S.K. et al. [31]	2018	ODNN	ELCAP	ELCAP	Abnormal vs. normal	96.2	94.2	N/A	94.5
Li, Li et al. * [17]	2018	CNN	LIDC-IDRI and NLST	Independent dataset	Multiple categories (overall)	N/A	N/A	N/A	N/A
Liao, Fangzhou et al. [23]	2019	CNN	LUNA16 and DSB17	DSB17	Cancer vs. no-cancer (scale)	N/A	N/A	87	81.4
Liu, Shuang et al. [32]	2017	CNN	NLST and ELCAP	NLST and ELCAP	Malign vs. benign	N/A	N/A	78	N/A
Liu, Xinglong et al. * [33]	2017	CNN	LIDC-IDRI	ELCAP	Multiple categories (overall)	N/A	N/A	N/A	90.3
Masood, Anum et al. [21]	2018	FCNN	LIDC-IDRI, RIDER, LungCT-Diagnosis, LUNA16, LISS, SPIE challenge dataset and Independent dataset	Independent dataset	Four stage categories (overall)	83.7	96.2	N/A	96.3
Nishio, Mizuho et al. [34]	2018	CNN	Independent dataset	Independent dataset	Benign, primary and metastic cancer (overall)	N/A	N/A	N/A	68
Onishi, Yuya et al. [35]	2018	DCNN	Independent dataset	Independent dataset	Malign vs. benign	N/A	N/A	84.1	81.7
Polat, Huseyin et al. [36]	2019	CNN	DSB17	DSB17	Cancer vs. no cancer	88.5	94.2	N/A	91.8
Qiang, Yan et al. [37]	2017	Deep SDAE-ELM	Independent dataset	Independent dataset	Malign vs. benign	84.4	81.3	N/A	82.8
Rangaswamy et al. [38]	2019	CNN	ILD	ILD	Malign vs. benign	98	94	N/A	96
Sori, Worku Jifara et al. [39]	2018	CNN	LUNA16 and DSB17	DSB17	Cancer vs. no cancer	87.4	89.1	N/A	87.8
Suzuki, Kenji * [19]	2009	MTANN	Independent dataset A	Independent dataset B	Malign vs. benign	96	N/A	N/A	N/A
Tajbakhsh, Nima et al. [20]	2017	CNN	Independent dataset	Independent dataset	Malign vs. benign	N/A	N/A	77.6	N/A
		MTANN	Independent dataset	Independent dataset	Malign vs. benign	N/A	N/A	88.1	N/A
Wang, Shengping et al. [40]	2018	CNN	Independent dataset	Independent dataset	PIL vs. IAC	88.5	80.1	89.2	84
Wang, Yang et al. [25]	2019	RCNN	Independent dataset	Independent dataset	Malign vs. benign	76.5	89.1	90.6	87.3
Yuan, Jingjing et al. * [41]	2017	CNN	LIDC-IDRI	ELCAP	Multiple categories (overall)	N/A	N/A	N/A	93.9
Zhang, Chao et al. * [42]	2019	CNN	LUNA16, DSB17 and Independent dataset(A)	Independent dataset(B)	Malign vs. benign	96	88	N/A	92

Studies marked with * are studies where test dataset was different from training dataset. Abbreviations: massive training artificial neural network (MTANN), convolutional neural network (CNN), deep neural network (DNN), lung image database consortium and image database resource initiative (LIDC-IDRI), the Dutch–Belgian randomized lung cancer screening trial (Dutch acronym; NELSON), multicentric Italian lung detection (MILD), laboratory of neuro imaging (LONI), early lung cancer action program (ELCAP), reference image database to evaluate therapy response (RIDER), Society of Photo-Optical Instrumentation Engineers (SPIE), lung nodule analysis 2016 (LUNA16), lung CT imaging signs (LISS), Kaggle data science bowl 2017 (DSB17), interstitial lung disease (ILD), Danish lung cancer screening trial (DLCST), automatic nodule detection 2009 (ANODE09), pre-invasive lesions (PIL), invasive adenocarcinomas (IAC).

**Table:** (**a**)

Author	Year	Sensitivity	Specificity
Jakimovski, Goran et al. [30]	2019	99.9	98.7
Lakshmanaprabu, S.K. et al. [31]	2018	96.2	94.2
Masood, Anum et al. [21]	2018	83.7	96.2
Polat, Huseyin et al. [36]	2019	88.5	94.2
Qiang, Yan et al. [37]	2017	84.4	81.3
Rangaswamy et al. [38]	2019	98	94
Sori, Worku Jifara et al. [39]	2018	87.4	89.1
Suzuki, Kenji et al. [19]	2009	96 *	N/A
Wang, Shengping et al. [40]	2018	88.5	80.1
Wang, Yang et al. [25]	2019	76.5	89.1
Zhang, Chao et al. [42]	2019	96 *	88 *

**Table:** (**b**)

Author	Year	AUC
Ciompi, Francesco et al. [28]	2015	84.7
Liao, Fangzhou et al. [23]	2019	87
Liu, Shuang et al. [32]	2017	78
Onishi, Yuya et al. [35]	2018	84.1
Tajbakhsh, Nima et al.(CNN) [20]	2017	77.6
Tajbakhsh, Nima et al.(MTANN) [20]		88.1
Wang, Shengping et al. [40]	2018	89.2
Wang, Yang et al. [25]	2019	90.6

**Table:** (**c**)

Author	Year	Accuracy
Alakwaa, Wafaa et al. [27]	2017	86.6
Chen, Sihang et al. [22]	2019	87.5
Ciompi, Francesco et al. [29]	2017	79.5 *
Jakimovski, Goran et al. [30]	2019	99.6
Lakshmanaprabu, S.K. et al. [31]	2018	94.5
Liao, Fangzhou et al. [23]	2019	81.4
Liu, Xinglong et al. [33]	2017	90.3 *
Masood, Anum et al. [21]	2018	96.3
Nishio, Mizuho et al. [34]	2018	68
Onishi, Yuya et al. [35]	2018	81.7
Polat, Huseyin et al. [36]	2019	91.8
Qiang, Yan et al. [37]	2017	82.8
Rangaswamy et al. [38]	2019	96
Sori, Worku Jifara et al. [39]	2018	87.8
Wang, Shengping et al. [40]	2018	84
Wang, Yang et al. [25]	2019	87.3
Yuan, Jingjing et al. [41]	2017	93.9 *
Zhang, Chao et al. [42]	2019	92 *

Results marked with * are from studies where test dataset was different from training dataset.
